# Three-Dimensional Printed Tooth Model with Root Canal Ledge: A Novel Educational Tool for Endodontic Training

**DOI:** 10.3390/dj11090213

**Published:** 2023-09-11

**Authors:** Rui Zhang, Renjie Tang, Sebastian Spintzyk, Yuting Tian, Yong Xiang, Yichen Xu, Tao Hu

**Affiliations:** 1 State Key Laboratory of Oral Diseases & National Center for Stomatology & National Clinical Research Center for Oral Diseases, Department of Preventive Dentistry, West China Hospital of Stomatology, Sichuan University, Chengdu 610041, China; 2 ADMiRE Research Center—Additive Manufacturing, Intelligent Robotics, Sensors and Engineering, School of Engineering and IT, Carinthia University of Applied Sciences, Europastraße 4, 9524 Villach, Austria; 3 State Key Laboratory of Oral Diseases & National Center for Stomatology & National Clinical Research Center for Oral Diseases, Department of Oral Prosthodontics, West China Hospital of Stomatology, Sichuan University, Chengdu 610041, China

**Keywords:** 3D printing, additive manufacturing, dental education, educational models, root canal therapy, ledge, endodontics

## Abstract

Ledge formation presents a significant challenge in endodontic treatment. Yet, there is still a lack of educational tooth models for hands-on practice. This study aimed to create and evaluate a tooth model for ledge management practice. A natural tooth with curved roots was collected for scientific use under ethics committee approval. Following initial root canal preparation, the tooth was scanned using micro-computed tomography (μCT) and 3D reconstructed. A K-file, created via computer-aided design (CAD), was partly inserted into the root canal wall of the 3D reconstructed tooth. By subtracting the K-file from the tooth, a tooth model with a root canal ledge was produced. The model was then 3D printed for a hands-on workshop. An eight-item Likert-scale questionnaire was administered to 20 postgraduate students and 10 endodontists to assess the model’s quality and training effectiveness. In addition, the success rate of bypassing and correcting the root canal ledge was documented. The feedback from both the students and experts was positive, and the results of the Mann–Whitney U test indicated no statistically significant differences found between the two groups (*p* > 0.05). The success rate of the students and the experts was 85% and 100%, respectively. In future applications, this novel tooth model is expected to address the existing gap in endodontic education and provide benefits for dental practitioners.

## 1. Introduction

In endodontic treatment, maintaining the original shape and path of the root canal is of paramount importance for a successful outcome [1,2]. However, one of the challenges often encountered in clinical practice is the creation of a “ledge”—a deviation from the original pathway of the root canal [3]. This procedural error has been identified as one of the most common complications in endodontics, significantly impacting the course of treatment and potentially altering the long-term prognosis of the tooth being treated [4].

A ledge is formed when the endodontic instrument deviates from the natural canal path, typically in curved or narrow canals, resulting in an artificial barrier on the canal wall [3,5]. The development of a ledge can prevent the instrument from reaching the apical stop, thus obstructing shaping, disinfection, and subsequent filling of the canal system to its working length. As a result, an uneliminated infection may contribute to the continuation of periapical periodontitis [6,7]. A graver concern is that the presence of a ledge can potentially misdirect root canal instrumentation, leading to possible lateral perforation of the tooth [8]. This erroneous path taken by the instrument not only compromises the integrity of the root structure but also poses a significant risk to the overall success of the endodontic procedure.

A clinical investigation performed by Greene et al. indicated that 46% of root canals shaped by dental students had been ledged [5]. Similarly, Kapalas et al. reported that 52% of canals treated by dental students resulted in ledge formation [9]. In comparison, when endodontists performed the procedure, the incidence of ledge creation reduced substantially to 33% [9]. This highlights the crucial role of a dentist’s experience and technical skill in preventing ledge formation. In addition to prevention, ledge management also holds significant importance [3]. Dental students should be trained to recognize, evaluate, bypass, and correct ledges once formed to avoid severe iatrogenic accidents. Although dental students can gain experience and make progress from their mistakes in clinical work, patients should not be the subject of practice with their health interests at stake. Currently, dental students’ understanding of ledges mostly comes from descriptions in textbooks and existing endodontic literature, highlighting a significant gap in the availability of highly realistic educational tools for pre-clinical practice.

The burgeoning landscape of digital technology unfolds novel avenues for the evolution and enhancement of dental education. Additive manufacturing, also known as 3D printing, is a cutting-edge technique that creates physical objects from digital designs by depositing the material of interest layer by layer until the final product is formed [10,11]. This process differs fundamentally from traditional subtractive manufacturing methods that remove materials, allowing complex and intricate designs to be created with minimal material waste and great precision [12]. At present, 3D printed training models have showcased promising prospects in enhancing the quality of dental education [13,14,15], but training models for ledge management are still lacking.

The present study aimed to establish a digital workflow to fabricate a realistic 3D printed tooth model with a root canal ledge for hands-on endodontic training. The benefits of the 3D printed tooth model were evaluated by postgraduate students and endodontic experts using a Likert-scale questionnaire. These models were expected to help dental students and practitioners gain experience in ledge management, thus filling the gap in endodontic education.

## 2. Materials and Methods

### 2.1. Model Fabrication

#### 2.1.1. Tooth Preparation

**Figure 1:**
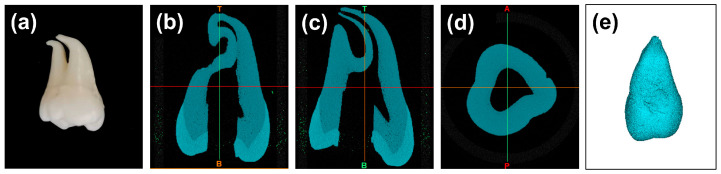
μCT scanning of the prepared tooth. (**a**) The prepared tooth subjected to μCT scanning; (**b**–**d**) represent the coronal, sagittal, and horizontal views of the 3D reconstructed tooth in Materialise Mimics, respectively; and (**e**) the 3D reconstructed tooth.

With approval from the Medical Ethics Committee of West China Hospital of Stomatology, Sichuan University (No. WCHSIRB-D-2021-211), an impacted mandibular third molar was extracted and collected for scientific use. The acquired tooth was free from caries and showed no signs of hard tissue loss. The roots were fully matured with closed apexes and exhibited a notable curvature in their apical third. After tooth extraction, the residual periodontal tissues were carefully removed from the roots using a sharp scalpel. The tooth was then soaked in 3% hydrogen peroxide for three days. After endodontic cavity preparation, the location of the root canal orifice was carefully detected, and the pulp was removed using a barbed broach (MANI Inc., Tochigi, Japan). Subsequently, the root canals were initially negotiated to their working length using a No. 10 K-file (Dentsply Maillefer, Ballaigues, Switzerland), and then enlarged and shaped using a No. 15 K-file (Dentsply Maillefer, Ballaigues, Switzerland). A 3% sodium hypochlorite solution (Longly, Wuhan, China) was intermittently used for irrigation to clear the debris (Figure 1a).

#### 2.1.2. Micro-Computed Tomography (μCT) Scanning

The prepared tooth was subjected to high-resolution micro-computed tomography (μCT) scanning using a μCT scanner (μCT45, Scanco Medical, Brüttisellen, Switzerland). The scanner was set up to capture images with a voxel size of 10 μm. To obtain a comprehensive view of the tooth’s internal structures, the scanning protocol included 250 projections over a 180° rotation. The tube diameter was set at 9/8 mm. After the scanning process, the resulting Digital Imaging and Communications in Medicine (DICOM) data were imported into Materialise Mimics (21.0, Materialise, Leuven, Belgium) and 3D reconstructed. Specifically, a grayscale threshold range between 1637 Hounsfield Units (HU) and 15,024 HU was applied to segment the tooth from the background. To further refine the model, a region-growing algorithm with 6-connectivity was utilized to select and aggregate voxels with similar density values. After ensuring the accuracy of the 3D model, it was exported in the standard tessellation language (STL) format for further use (Figure 1b–d).

#### 2.1.3. Computer-Aided Design (CAD) of K-File

**Figure 2:**
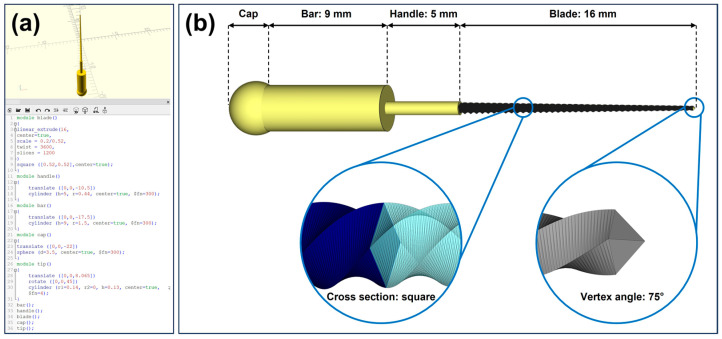
CAD model of a No. 20 K-file. (**a**) The K-file was created in OpenSCAD using its scripting language, and (**b**) schematic illustration of the designed K-file. The K-file consists of four parts: the blade, the handle, the bar, and the cap. The tip of the blade is a four-sided pyramid with a vertex angle of 75°, and the cross-sectional shape of the blade is square.

A No. 20 K-file was designed using the CAD software (OpenSCAD, 2021.01, http://www.openscad.org/, accessed on 23 March 2023) through programming (Figure 2a). For readers keen on delving deeper into the CAD process, the programming code is available in Appendix A. Overall, the design of the K-file was achieved by modeling and assembling four parts, i.e., the blade, the handle, the bar, and the cap. As shown in Figure 2b, the blade design was achieved by linearly extruding a square with a side length of 0.52 mm over a distance of 16 mm, with a 3600-degree twist, which was then uniformly scaled down to a square with a side length of 0.2 mm. The tip of the blade was designed as a four-sided pyramid with a vertex angle of 75°. The handle connecting to the blade was created as a cylinder with a height of 5 mm and a radius of 0.44 mm. The bar was designed similarly to the handle, as a cylinder with a height of 9 mm and a radius of 1.5 mm. The cap, designed as a sphere with a diameter of 3.5 mm, was positioned to partially overlap with the bar. The completed CAD design of the K-file was saved in the STL format for subsequent use.

#### 2.1.4. CAD of the Tooth Model with Root Canal Ledge

**Figure 3:**
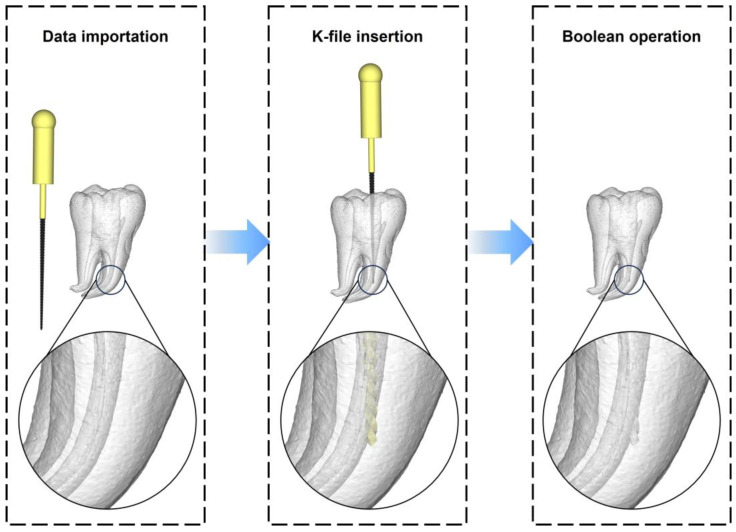
CAD of the tooth model with root canal ledge. Left: the STL data of the 3D reconstructed tooth and the K-file were imported into the Materialise Magics software. Middle: the tip of the K-file was partly inserted into the outer wall of the curved section of a root canal. Right: the tooth model with root canal ledge was obtained by subtracting the K-file from the 3D reconstructed tooth model using Boolean operations.

The STL files of the 3D reconstructed tooth, as described in Section 2.1.2, and the K-file, as described in Section 2.1.3, were both imported into the Materialise Magics software (25.0, Materialise, Leuven, Belgium). Subsequently, the K-file was positioned in one of the curved root canals of the 3D reconstructed tooth using the translate and rotate functions. The tip of the K-file was partly inserted into the outer wall of the curved section of the root canal, mimicking the formation of a ledge. Following this, Boolean operations were applied to subtract the K-file model from the 3D reconstructed tooth, resulting in a tooth model with a root canal ledge. The model was then saved in the STL format for subsequent additive manufacturing (Figure 3).

#### 2.1.5. Additive Manufacturing

**Figure 4:**
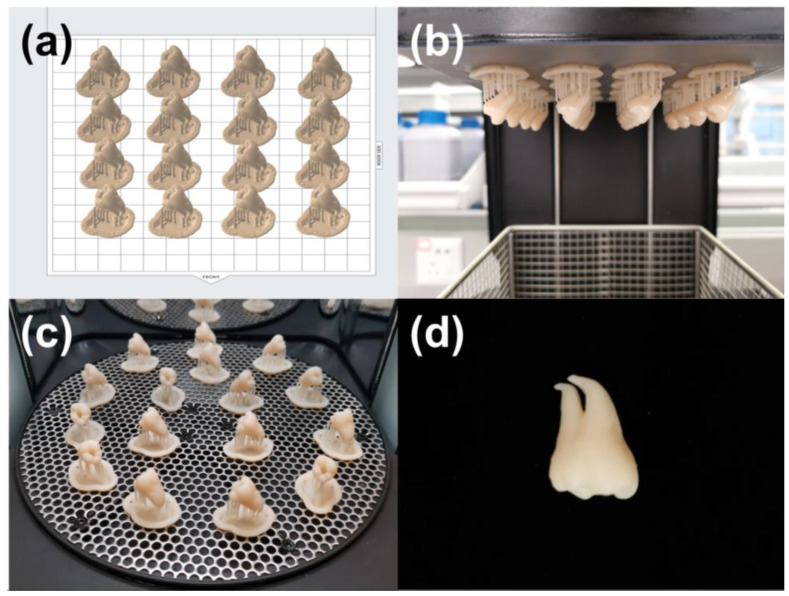
Additive manufacturing of the tooth model used for ledge management practice. (**a**) The printing direction and layout on the build platform; (**b**,**c**) show post-cleaning and post-curing procedures, respectively; and (**d**) the 3D printed tooth model.

The model, obtained as described in Section 2.1.4, was 3D printed with no scaling using a stereolithography (SLA) printer (Form 3B, Formlabs, Somerville, MA, USA), with a 50 μm layer thickness, and a photopolymer resin (Model V3, Formlabs, Somerville, MA, USA). Figure 4a illustrates the printing direction and layout. To ensure printing accuracy, the angle between the tooth’s long axis and the build platform was set to approximately 45°. A total of 16 models were printed simultaneously for 2 h and 48 min. Following the printing process, the models were cleansed with isopropyl alcohol (IPA, Chron chemicals, Chengdu, China) for a duration of 10 min using a post-cleaning device (Form Wash, Formlabs, Somerville, MA, USA). Afterward, the models underwent a post-curing process under 405 nm blue light at a temperature of 60 °C for a period of 5 min in a post-curing device (Form Cure, Formlabs, Somerville, MA, USA). Upon completion of the post-curing process, the support structures were carefully detached using a specialized finishing kit provided by Formlabs. Any remnants of the support structures were meticulously trimmed using a sharp scalpel to ensure a clean and precise final model.

### 2.2. Model Evaluation

To evaluate the fabricated training models, we organized a hands-on workshop with voluntary participation. According to the minimum sample size determined by the n-Star method [16], 30 individuals, including 20 postgraduate students and 10 endodontists, participated in this hands-on course. The students had already completed their basic theoretical study in endodontic treatment and performed at least 50 root canal treatments on patients. To maintain the objectivity of the evaluation, the students were assured that their performance during the evaluation would not have any influence on their academic scores.

At the outset of the workshop, we conducted a brief review for the students on the concept of ledge formation and the reasons for its occurrence. Subsequently, the techniques for ledge management were outlined, which focused on (1) recognizing ledges, which is characterized by the loss of the typical tactile sensation of the endodontic instrument’s tip as the feeling of being held by the canal walls transitioning into a sensation of hitting against a canal wall; (2) bypassing ledges, which is achieved by pre-bending the endodontic instrument to facilitate the tip’s gentle slide along the inner surface of the curved canal, thereby assisting in bypassing the ledge and reaching the working length; and (3) correcting ledges, which involves positioning and maintaining the file tip apical to the ledge, applying gentle pressure with the file against the outer wall where the ledge is located, and then moving the file in and out over a short distance.

Next, the participants were asked to practice the techniques mentioned above using the 3D printed models. The benefits of the model were assessed via a questionnaire (Table 1) created by the Teaching and Research Section of Preventive Dentistry at the university using SurveyStar (Ranxing Information Technology Co., Ltd., Changsha, China). The participants were asked to rate 8 items on a Likert scale ranging from 1 to 5 in the questionnaire (1 = excellent, 2 = good, 3 = satisfactory, 4 = adequate, 5 = poor). The first three items pertained to the quality of the model, while the remaining five items assessed the educational effect of the tooth model. In addition, free comments from the participants were also collected. Based on the results of the questionnaire, we calculated the frequencies and mean values (Ø) of ratings. To objectively evaluate the difficulty of the 3D printed training model, the success rate of the students and the experts in bypassing and correcting the root canal ledge was documented. Furthermore, for participants who successfully bypassed the root canal ledge, the time they used was recorded.

### 2.3. Statistical Analysis

The internal consistency among the items in the questionnaire was examined via Cronbach’s alpha test using the SPSS statistics software (26.0, IBM, New York, NY, USA). An obtained Cronbach’s alpha value greater than 0.7 was considered indicative of good reliability. The differences in ratings between the students and experts were examined using the Mann–Whitney U test. The time spent by the students and experts on bypassing the ledge was compared using the Student’s t-test. GraphPad Prism (9.5.1, GraphPad Software, San Diego, CA, USA) was used for statistical analyses. A *p*-value of 0.05 was used to determine statistical significance.

## 3. Results

### 3.1. Three-Dimensional Printed Model for Endodontic Training

**Figure 5:**
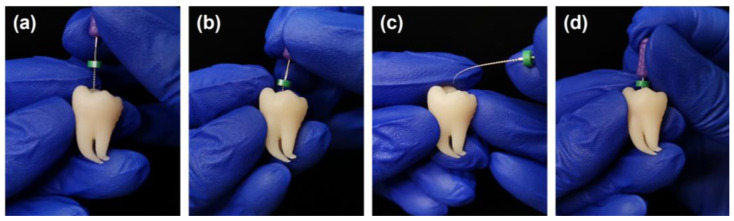
Applying the 3D printed tooth model for endodontic training: (**a**) root canal negotiation; (**b**) ledge detection; (**c**) instrument pre-bending; and (**d**) ledge bypassing and correction.

As shown in Figure 5, the 3D printed tooth model could be successfully applied in endodontic training. The model incorporated a highly realistic root canal system for endodontic practice (Figure 5a). When a straight K-file was inserted into the curve portion of the root canal, resistance force and tactile change caused by the ledge could be experienced (Figure 5b). By pre-bending, the instrument could bypass the ledge and reach the working length (Figure 5c,d).

### 3.2. Evaluation of the Model

All participants completed the questionnaire, resulting in a 100% response rate. The questionnaire demonstrated good internal consistency, with a Cronbach’s alpha value of 0.74. For all eight items in the questionnaire, the students and experts provided similar ratings. Accordingly, no statistically significant differences were found between the student and expert groups according to the results of the Mann–Whitney U test (*p* > 0.05, Table 2).

**Figure 6:**
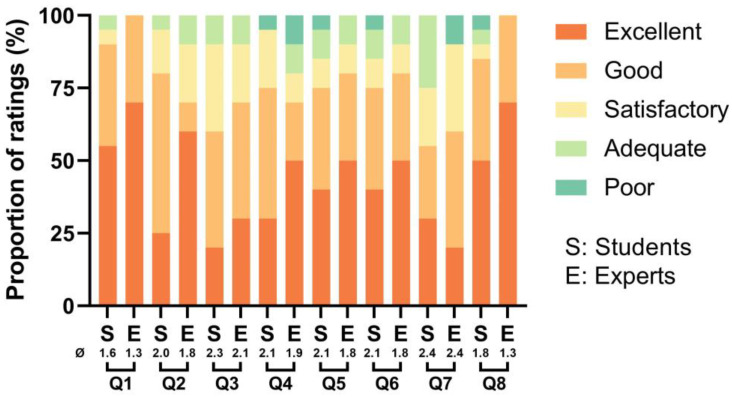
The proportion of ratings for each item evaluated by the students and experts. Q1–Q8 correspond to the items listed in the questionnaire (Table 1). The symbol “Ø” represents the mean value for each group.

As illustrated in Figure 6, both experts and students gave relatively positive evaluations concerning the quality of the model. They rated the realism of the 3D printed model in simulating the anatomical structure of a natural tooth as good to excellent (Q1: Ø = 1.6 for students and Ø = 1.3 for experts), the realism of the model in simulating a root canal ledge as good to excellent (Q2: Ø = 2.0 for students and Ø = 1.8 for experts), and its material texture replication of a natural tooth as satisfactory to good (Q3: Ø = 2.3 for students and Ø = 2.1 for experts).

Concerning the educational effect of the model, both students and experts agreed that practicing with the 3D printed model was beneficial for recognizing (Q4: Ø = 2.1 for students and Ø = 1.9 for experts), bypassing (Q5: Ø = 2.1 for students and Ø = 1.8 for experts), and correcting (Q6: Ø = 2.1 for students and Ø = 1.8 for experts) root canal ledges to avoid potential lateral perforation (Q7: Ø = 2.4 for both students and experts). Lastly, both groups of participants believed that incorporating the 3D printed model into future endodontic training could potentially achieve “good” to “excellent” educational outcomes (Q8: Ø = 1.8 for students and Ø = 1.3 for experts).

**Figure 7:**
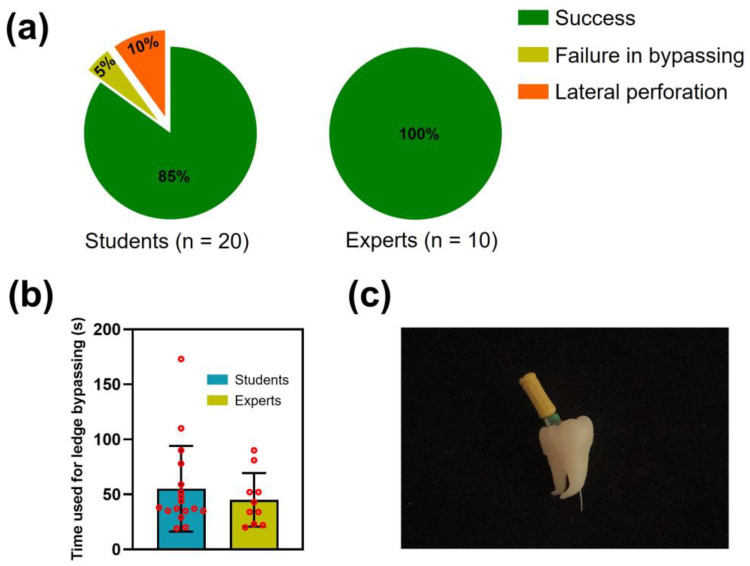
Objective evaluations of the 3D printed tooth model used for ledge management practice. (**a**) Success rate for the students and experts in bypassing and correcting the ledge. (**b**) Time taken by the students and experts to bypass the ledge (Mean ± SD). Red circles represent individual participant data. (**c**) Representative photograph of a model showing lateral perforation during practice.

As depicted in Figure 7a, among the student group, 17 participants successfully bypassed and corrected the ledge (85%). One student failed to bypass the ledge and ultimately ceased the attempt (5%), and two students experienced lateral perforation of the root canal during their practice (10%, Figure 7c). In contrast, the expert group achieved a 100% success rate. For those who bypassed the ledge, the students took an average time of 55.24 ± 38.92 s, while the experts took an average time of 45.10 ± 24.27 s. Although no statistically significant difference was detected between the two groups (*p* > 0.05), the student group exhibited a higher standard deviation, which could be further confirmed by the data points deviating significantly from the mean value (Figure 7b).

## 4. Discussion

Given the notable absence of endodontic training models specifically designed for ledge management practice, this study combined μCT scanning, CAD, and additive manufacturing to establish a digital workflow for creating tooth models with a root canal ledge. Undoubtedly, extracted teeth serve as irreplaceable resources for preclinical endodontic education, providing students with genuine human tissue for hands-on practice [17]. In addition to extracted teeth, there are also commercially available models for endodontic training, such as resin teeth and resin blocks [18]. These models can serve as supplements to address the shortage of extracted teeth, thereby meeting the vast demand for preclinical practice among dental students. Compared to extracted teeth, one advantage of these models is their reproducibility [19], which could allow students to practice repeatedly with the same model, or could ensure consistent difficulty levels for exams. However, pre-made models are confined to the specific types provided by the manufacturer, thus failing to represent the diversity and peculiarities of human teeth. This drawback may severely hamper students’ cognition and understanding in endodontic learning. With the rapid advancement of digital dental technologies in recent years, 3D printing technologies hold promise in resolving this issue by customized manufacturing. To date, a number of 3D printed tooth models have been developed for preclinical practice, such as models for crown preparation [14], caries removal [15,20], and root canal therapy [21,22], driving forward the progression of dental education.

**Figure 8:**
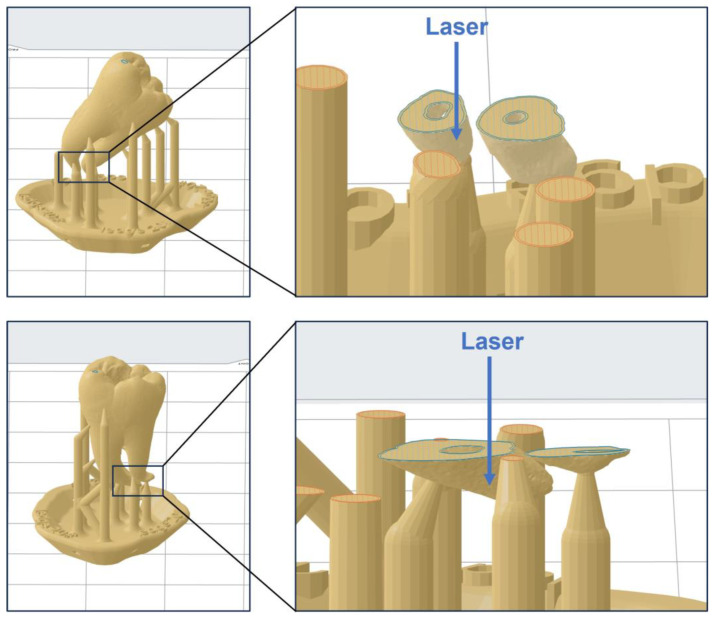
Schematic illustration of the impact of printing direction on the accuracy of the model’s apical part. The area circled in blue represents the layers in the 3D printing of the model (left images: the final layers of 3D printing; right images: layers from the 3D printing of the apical part). The area circled in red denotes the layers in the 3D printing of the support structure.

In this study, additive manufacturing serves as the pivotal technology for model fabrication. This is due to the working principle of 3D printing, which allows the fabrication of complex structures. The root canal system is one of such examples, especially when the roots are significantly curved [22,23]. However, the fabrication process in this study still faced challenges because the root canal near the apex is extremely narrow. Even though we adopted a relatively accurate 3D printing technology (SLA) [24], blockage at the apical part of the root canal was prone to occur. To address this issue, we utilized an accurate material (Model V3) provided by the manufacturer. More importantly, the printing direction was adjusted by setting the angle to approximately 45°. As illustrated in Figure 8, when the model is printed vertically, the curing laser emitted by the 3D printer may penetrate the thin root canal wall at the apex, causing the liquid resin inside the root canal to solidify, thus leading to blockage at the apex. This issue is particularly prone to occur when using transparent materials. In contrast, printing the model at a 45° angle can circumvent this problem.

In the present study, we employed the programming-based CAD software (OpenSCAD) for the 3D modeling of the K-file. This approach was adopted because K-files are diminutive in size. Due to the limited scanning accuracy [25,26], current 3D scanners may not precisely capture the 3D details of a K-file, especially the intricate structure of the blade. In contrast to interactive modelers, the programming-based CAD software offers greater control over the entire modeling process by allowing direct adjustments to the programming code. During our 3D modeling process, the blade design of the K-file was relatively challenging as it required simultaneous consideration of the diameter, taper, cross-section, and tip. With the designed K-file and the 3D reconstructed tooth, we virtually created a ledge using Boolean subtraction. It might be easier and more clinically relevant to manually create a ledge using real K-files and an extracted tooth for subsequent μCT scanning and 3D reconstruction. However, the virtual creating process of a ledge could be more efficient in dynamically adjusting the difficulty level of the model. By moving the K-file inserted into the root canal wall, one can directly modify the location, direction, depth, and size of a ledge on demand.

**Figure 9:**
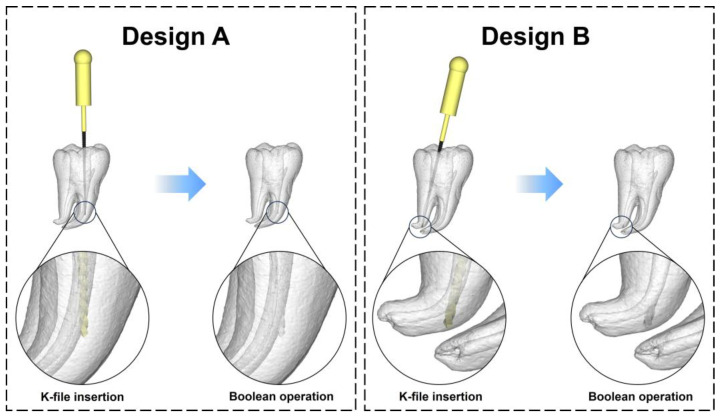
Schematic illustration of the influence of model design on the difficulty level of the model. Design A is the model design used in this study. Design B is an example of a model design with greater difficulties for practicing.

The results of the subjective evaluation by the students and endodontists indicated that the fabricated model exhibited good performance in terms of model quality and educational effect. The two relatively lower-rated items in the questionnaire were Q3 and Q7. We analyzed the potential reasons to guide further improvements of the model. Regarding Q3, the relatively lower rating could be attributed to the inferior hardness and wear resistance of the resin compared to that of natural teeth [27]. For future enhancements, more durable 3D printing resins could be considered for model fabrication. In terms of Q7, a potential explanation might be that practicing with the tooth model cannot effectively replicate real-world treatment situations. The participants knew the shape and curvature of the root prior to practice, which might decrease the difficulties in sensing root canals by instruments. For future applications, mounting the 3D printed tooth model onto a manikin simulator may yield better educational outcomes. The difference in success rates of bypassing and correcting the root canal ledge between the experts and students highlights the crucial role of dentists’ experience and skills in ledge management (Figure 7a). As an educational tool, the 3D printed model prepared in this study may possess an insufficient difficulty. This is evidenced by the students’ relatively high success rate (85%) and the lack of statistical difference in the time taken to bypass the ledge between the students and experts (Figure 7b). Since patience is crucial in ledge management, we did not set a limit on the number of attempts or on the time taken to bypass the ledge. To further improve the discriminative capability of the model, especially for examination purposes, such success criteria could be set. It should be clarified that, instead of introducing a specific product, the present study aimed to develop a method for creating tooth models used for ledge management practice. Utilizing this method, dental educators can customize models with varying difficulty levels for different students because the location, direction, depth, and size of the ledge can be modified on demand. Figure 9 presents an example of a model design whose difficulty level might be more suitable for experienced students (Design B). However, how to design appropriate models for students with varying proficiency levels requires further research for a comprehensive elucidation.

The primary limitation of this study is the absence of a control group for comparative analysis, given the scarcity of commercially available models designed specifically for ledge management training. Table 3 enumerates several common endodontic training models on the market. It is evident that the majority of these models, whether resin teeth or resin blocks, are tailored for beginners in endodontics, focusing on foundational techniques such as access cavity preparation, root canal preparation, and root canal filling. Models that enable the practice of more challenging endodontic techniques, such as ledge management, removing fractured instruments, and repairing lateral perforation, are still in great need.

Due to the considerable incidence (33%) of ledge formation among endodontists [9], this study included 10 experts for model evaluation alongside the students. The results of this study show that both groups had similar perceptions about the 3D printed training model, which is worth promoting in future endodontic education (Q8) to benefit dental practitioners.

## 5. Conclusions

By utilizing μCT scanning, CAD, and additive manufacturing, this study developed a digital workflow for fabricating a tooth model specifically designed for practicing ledge management in endodontic education. Both students and experts provided positive feedback regarding the quality of the model and its effectiveness in endodontic training. This novel method is expected to fill the gap in endodontic education, thus offering substantial benefits to dental practitioners.

## Figures and Tables

**Table 1:** Questionnaire for evaluating the 3D printed tooth model.

Category	Item Number	Content
Model quality	Q1	The realism of the 3D printed model in simulating the anatomical structure of natural teeth.
	Q2	The realism of the 3D printed model in simulating root canal ledges.
	Q3	The realism of the 3D printed model in simulating the material texture of natural teeth.
Educational effect	Q4	The extent to which practicing with the 3D printed model aids in recognizing root canal ledges.
	Q5	The extent to which practicing with the 3D printed model aids in bypassing root canal ledges.
	Q6	The extent to which practicing with the 3D printed model aids in correcting root canal ledges.
	Q7	The extent to which practicing with the 3D printed model aids in managing ledges to avoid lateral perforation.
	Q8	The degree of potential educational effect of applying the 3D printed model in future endodontic training.

**Table 2:** Comparative analysis of ratings between the students and experts using the Mann–Whitney U test.

	Q1	Q2	Q3	Q4	Q5	Q6	Q7	Q8
*p*-value ^1^	0.40	0.36	0.57	0.64	0.64	0.64	0.99	0.26
S/E ^2^	328/137	330/135	323/142	321/144	321.5/143.5	321.5/143.5	311/154	334.5/130.5
U statistic	82	80	87	89	88.5	88.5	99	75.5

^1^ The reported *p*-values are two-tailed. ^2^ S/E represents the sum of ranks in the student/expert group.

**Table 3:** Common endodontic training models on the market.

Manufacturer	City/Country	Product No.	Model Type	Training Purpose
VDW GmbH	Munich/Germany	V040247000000	Resin teeth and resin blocks	Root canal preparation and electronic length determination
Navadha	Mumbai/India	ADC07-3	Resin teeth	Access cavity preparation, root canal preparation, and root canal filling
NISSIN DENTAL PRODUCTS INC.	Kyoto/Japan	S/E/B Series	Resin teeth and resin blocks	Access cavity preparation, root canal length measurement, root canal preparation, and root canal filling
Rogin Medical Co., Ltd.	Shenzhen/China	R38-0896	Resin blocks	Root canal preparation and root canal irrigation

## Data Availability

All data from this study have been provided in the Results Section and can be obtained from the corresponding author upon reasonable request.

## Appendix A. Programming Code of a No. 20 K-File in OpenSCAD Software

module blade()
{
linear_extrude(16,
center=true,
scale=0.2/0.52,
twist=3600,
slices=1200
)
square ([0.52,0.52], center=true);
}
module handle()
{
translate ([0,0,-10.5])
cylinder (h=5, r=0.44, center=true, $fn=300);
}
module bar()
{
translate ([0,0,-17.5])
cylinder (h=9, r=1.5, center=true, $fn=300);
}
module cap()
{
translate ([0,0,-22])
sphere (d=3.5, center=true, $fn=300);
}
module tip()
{
translate ([0,0,8.065])
rotate ([0,0,45])
cylinder (r1=0.14, r2=0, h=0.13, center=true, $fn=4);
}
bar();
handle();
blade();
cap();
tip();
		
